# Correction: Hsieh et al. Single-Grain Gate-All-Around Si Nanowire FET Using Low-Thermal-Budget Processes for Monolithic Three-Dimensional Integrated Circuits. *Micromachines* 2020, *11*, 741

**DOI:** 10.3390/mi16050537

**Published:** 2025-04-30

**Authors:** Tung-Ying Hsieh, Ping-Yi Hsieh, Chih-Chao Yang, Chang-Hong Shen, Jia-Min Shieh, Wen-Kuan Yeh, Meng-Chyi Wu

**Affiliations:** 1 Institute of Electronics Engineering, National Tsing Hua University, Hsinchu 30013, Taiwan; tim.hsieh@latticesemi.com (T.-Y.H.); mcwu@ee.nthu.edu.tw (M.-C.W.); 2 National Applied Research Laboratories, 3F, No. 106, Ho Ping E. Rd., Sec. 2, Taipei City 10622, Taiwan; 3 Taiwan Semiconductor Research Institute, No. 26, Prosperity Road 1, Hsinchu 30013, Taiwan; ping-yi.hsieh@imec.be (P.-Y.H.); chshen@narlabs.org.tw (C.-H.S.); jmshieh@narlabs.org.tw (J.-M.S.); 1305023@narlabs.org.tw (W.-K.Y.)

## 1. Error in Figure 2

In the original publication [1], there was a mistake in Figure 2 as published. In the paper, we demonstrated monolithic 3D GAA-FETs using low thermal budget processes that have been proved and partially disclosed for monolithic 3D stacking in past conference papers [2,3]. In Figure 2a, the original FIB image was selected from IEDM2016 to represent the sequential 3DIC. It should be the same as the image we published in IEDM2016. However, it seems that it was a mirror image in this paper. After discussion, this might be due to an editing error when we labeled the text in the image and rearranged the figure. Unfortunately, the original FIB file was lost. To correct this error, we prepared another FIB image (supporting FIB image) instead and modified the figure caption accordingly (Figure 2 and caption). The corrected Figure 2 appears below. 

## 2. Error in Figure 6

In the original publication, there was a mistake in Figure 6 as published. The TEM images were cut, and the scale bar was redrawn to present clearer information in this paper. However, the numbers of the scale bar in this paper seem incorrect compared to the image we published in Figure 2b in 2015 IEDM [3]. This might be due to an unconscious editing error when we rearranged the image and edited Figure 6b. To correct this issue, we prepared another TEM image of the Si FET (supporting TEM image) using the same silicide process conditions and device structure. The corrected Figure 6 appears below. The authors state that the scientific conclusions are unaffected. This correction was approved by the Academic Editor. The original publication has also been updated.

## 3. Text Correction

There was an error in the original publication. The last sentence on page 3 of 12 in our *Micromachines* paper (doi:10.3390/mi11080741) “A two tier monolithic 3D-IC can be simply realized by repeating the whole low-thermal budget processes (T_sub_ < 400 °C) (Figure 2a,b).” needs a more detailed description.

A correction has been made to the last sentence on page 3 of 12 in our *Micromachines* paper (doi:10.3390/mi11080741):

A two-tier monolithic 3D-IC can be simply realized by repeating the whole low-thermal budget processes (T_sub_ < 400 °C) as we have shown in IEDM 2016 [2]. Figure 2a shows a two-tier monolithic 3D-IC having a metal interconnect to connect the top and bottom Si FETs. By using the proposed single-grain GAA Si NW FETs, the monolithic 3DIC will have better system performance, as illustrated in Figure 2b.

## 4. Missing Citation

In the original publication, reference 40 was not cited [3]. The citation has now been inserted on page 8 of 12, line 6, and should read:

The mixing of Ni_2_Si and Si occurs via liquid phase diffusion [38], which leads to a uniform NiSi film without heterogeneous aggregation and spike at the interface between the NiSi and the Si, which is confirmed in Figure 6b,c [40].

The authors state that the scientific conclusions are unaffected. This correction was approved by the Academic Editor. The original publication has also been updated.

## Figures and Tables

**Figure 2:**
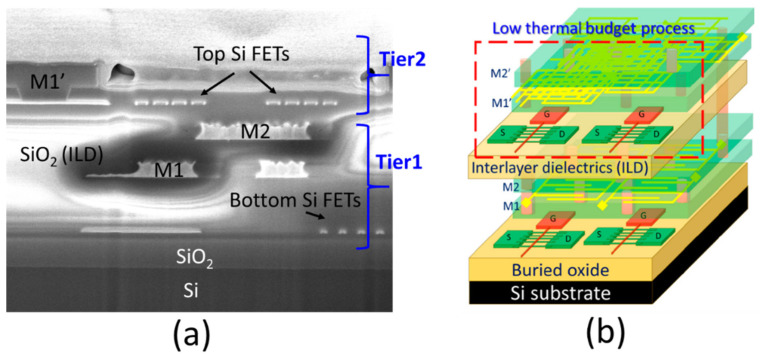
(**a**) FIB image of a 3D sequential integration with two stacking tiers and metal interconnects. (**b**) Schematic illustration of a monolithic three-dimensional integrated circuit (3DIC) using single-grain GAA Si NW FETs.

**Figure 6:**
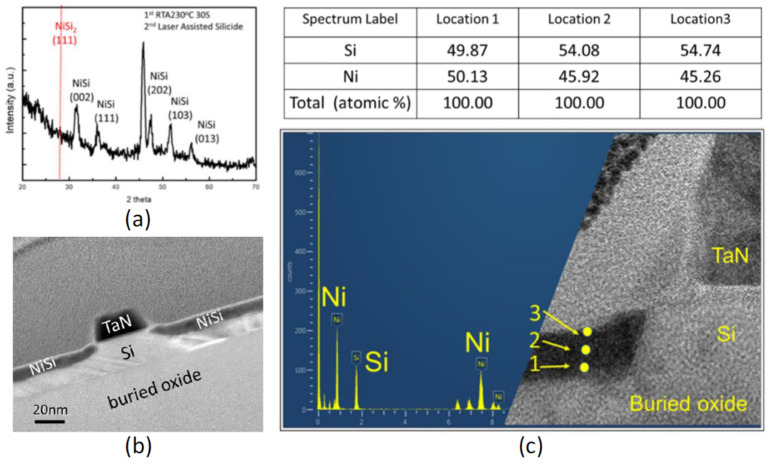
(**a**) The XRD patterns of the nickel silicide formed by the hybrid laser-assisted salicidation; (**b**) a TEM image of a Si FET with a flat and uniform NiSi film in the source and drain region; (**c**) energy-dispersive X-ray spectroscopy (EDS) information of the NiSi film in the source and drain region at various depths.
